# Four Cases of Pediatric Photokeratitis Present to the Emergency Department After Watching the Same Theater Show

**DOI:** 10.4274/tjo.67625

**Published:** 2015-10-05

**Authors:** Mehmet Serhat Mangan, Ceyhun Arıcı, Eray Atalay, Burak Tanyıldız, Faik Oruçoğlu

**Affiliations:** 1 Okmeydanı Education and Research Hospital, Clinic of Ophthalmology, İstanbul, Turkey; 2 İstanbul University Cerrahpaşa Faculty of Medicine, Department of Ophthalmology, İstanbul, Turkey; 3 Kars State Hospital, Clinic of Ophthalmology, Kars, Turkey; 4 İstanbul University İstanbul Faculty of Medicine, Department of Ophthalmology, İstanbul, Turkey; 5 Birinci Eye Hospital, İstanbul, Turkey

**Keywords:** Photokeratitis, ultraviolet, Cornea

## Abstract

We report four consecutive cases of photokeratitis that presented to the emergency department, interestingly after having watched the same theatre performance in the same school. The patients’ ages (3 male, 1 female) ranged from 9 to 13 years. All patients presented with similar complaints consisting of pain, tearing, foreign body sensation, photophobia and blurred vision in both eyes. Patients reported watching a theatre performance in the same school approximately 4 hours before symptom onset. On slit-lamp examination, conjunctival injection and corneal punctate epithelial erosions were observed in the interpalpebral zone in both eyes. On fundus examination, no pathology was observed in the vitreous, posterior pole or peripheral retina. All cases were treated with topical antibiotics and lubricant eye drops. Corneas were clear two days later in the control visit. In this case report, exposure to ultraviolet light from high-power lamps used in the theatre was proposed as a possible cause of corneal epithelial cell damage and subsequent photokeratitis.

## INTRODUCTION

Ultraviolet (UV) radiation between 200 and 400 nm, which comprises the UV-A, UV-B and UV-C wavelengths, has both beneficial and harmful effects on the human body. A strong relationship between UV radiation exposure and the eyelid cancers basal cell carcinoma and squamous cell carcinoma, photokeratitis, climatic droplet keratopathy, pterygium and cortical cataract development has been found, while the relationship between UV radiation and pinguecula, nuclear and posterior subcapsular cataract, ocular surface squamous neoplasia, ocular melanoma and age-related macular degeneration is limited.^1,2^

Photokeratitis is a clinical entity characterized by damage to the corneal epithelium due to exposure to UV light from natural or artificial sources.^3^ The cause is frequently occupational, and symptoms including photophobia, foreign body sensation, tearing and blurred vision typically appear 4 to 12 hours after UV light exposure. Characteristic clinical findings are conjunctival injection and areas of punctate erosion in the corneal epithelium, especially in the interpalpebral zone. The condition resolves within 24 to 48 hours with symptomatic treatment. There are case reports of photokeratitis developing in response to mercury vapor or metal halide lamps.^4,5,6,7,8,9,10,11,12^ Furthermore, cases of photokeratitis development due to the lighting used in gymnasiums have been reported.^13^ This article presents four patients who sequentially presented to emergency services with ocular complaints and were diagnosed with photokeratitis after watching a show in the same school theater.

## CASE REPORT

**Figure 1:**
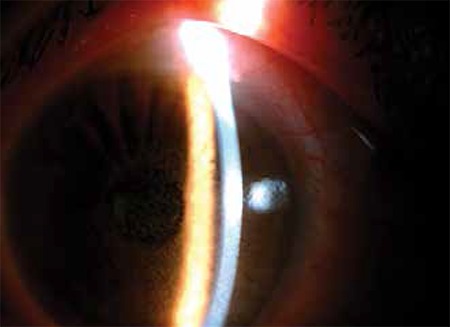
A photograph of the anterior segment showing severe punctate erosion of the corneal epithelium and conjunctival injection

**Figure 2:**
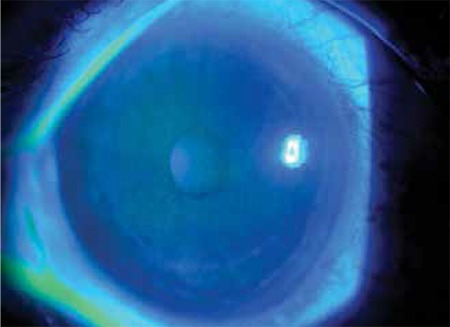
A photograph of the anterior segment under cobalt-blue light after staining with fluorescein showing corneal epithelial erosion, especially in the interpalpebral zone

Four pediatric patients between the ages of 9 and 13 (3 male, 1 female) sequentially presented to emergency services with complaints of bilateral ocular pain, foreign body sensation, tearing, photophobia and blurred vision within one hour of one another. Slit-lamp examination revealed severe punctate erosion and conjunctival injection in both eyes of all patients (Figure 1). Corneal epithelial erosion, especially in the interpalpebral zone, was better visualized with cobalt-blue light after staining with fluorescein (Figure 2). Fundus examination was normal in both eyes of all patients. Topical netylmicine drops (four times daily) and artificial tear drops (four times daily) were prescribed. At the day 2 follow-up appointment, all patients had clear corneas on slit-lamp examination. Common among the medical histories provided by the patients’ families was that the patients had watched the same show in the same school theater approximately 4 hours before symptom onset, and it was later learned that a light show had been intermittently aimed directly at the children.

## DISCUSSION

This study reports four pediatric cases in which ultraviolet radiation from lights during a theater show resulted in photokeratitis. Photokeratitis related to acute UV exposure causes severe ocular symptoms, resulting in emergency room admission. Although it is debatable as a cause of cataract development, in animal studies cataract development has been reported following acute UV exposure.^14^ Due to the possible risks, the necessity of protection against UV exposure, especially for children, is emphasized and the use of glasses or contacts that attenuate the effects of UV light is recommended in the USA.^15^

One study reported that UV light from metal halide lamps in gymnasiums had caused photokeratitis, especially in children, as we also showed in this report. The UV-B radiation emitted from these lamps causes photokeratitis. Ultraviolet wavelengths are generally absorbed by the cornea and conjunctiva, thereby causing keratitis and conjunctivitis. If the wavelength is sufficiently powerful, as in an ultraviolet laser, it can also reach the lens. Superficial punctate keratitis frequently develops. Although with direct thermal damage there is no latent period between exposure and the onset of clinical symptoms, with UV-related damage there is a latent period of 3-4 hours, as in the cases in this report. Corneal epithelium cell death begins 2-3 hours after exposure and subsequently blinking contributes to spontaneous fragmentation and results in symptom onset.

Different wavelengths of UV light are divided into three basic groups: UV-A (400-320 nm), UV-B (320-290 nm) and UV-C (290-200 nm). While UV-A causes photosensitivity reactions, UV-B gives rise to sunburn and skin cancer. UV-C has germicidal properties as well as causing skin cancer.

There are several simple precautions that can be taken to limit exposure to UV radiation. Among these are spending as little time as possible in environments with high intensity UV radiation, avoiding the sun between the hours of 10:00 and 14:00, wearing wide-brimmed hats and applying SPF 15 or higher sunscreen in sunny conditions, and using ski goggles, UV-protective hydrogel or hard gas permeable contact lenses, and sunglasses. Furthermore, the inspection of lighting systems in public areas and the legislative regulation of this issue are of great importance. With this case report, we aimed to emphasize that lamps that emit UV light can cause ocular damage and should not be used in public places.
